# Predictors of post-stroke depression: Validation of established risk factors and introduction of a dynamic perspective in two longitudinal studies

**DOI:** 10.3389/fpsyt.2023.1093918

**Published:** 2023-02-13

**Authors:** Simon Ladwig, Katja Werheid, Martin Südmeyer, Matthias Volz

**Affiliations:** ^1^Clinical Neuropsychology and Psychotherapy, Department of Psychology, Faculty of Psychology and Sport Science, Universität Bielefeld, Bielefeld, Germany; ^2^Department of Neurology, Klinikum Ernst von Bergmann, Potsdam, Germany; ^3^Department of Neurology, Heinrich-Heine-Universität, Düsseldorf, Germany; ^4^Clinical Psychology I, Department of Psychology, Faculty of Human Sciences, Universität Kassel, Kassel, Germany

**Keywords:** stroke, depression, dynamic risk factors, multivariable analysis, prospective longitudinal

## Abstract

**Introduction:**

Cerebral insults lead in many cases not only to cognitive impairment but also to disturbed emotionality. After stroke, one in three survivors develops a depression which impacts quality of life and rehabilitation. Meta-analyses have identified five main predictors of post-stroke depression (PSD): history of mental disorder, stroke severity, physical disability, cognitive impairment, and social support. However, these five established variables have never been conjointly investigated in a sample of stroke survivors. Therefore, their independent predictive values remain unclear. Moreover, predictors are most often used as time-invariant factors (status scores), neglecting the intraindividual dynamics after stroke.

**Methods:**

Our study analyses the data of two prospective longitudinal studies, investigating stroke survivors from two rehabilitation hospitals (*N*_1_ = 273) and one acute care hospital (*N*_2_ = 226). Baseline assessments included the five established predictors and depressive symptoms. After 6 months, depressive symptoms were reassessed in both studies (*n*_1_ = 176, *n*_2_ = 183), and physical disability and social support were reassessed in study 2. The predictivity of the five predictors and the additional predictivity of intraindividual dynamics for PSD were examined in multiple linear regression analyses.

**Results:**

History of mental disorder was a risk factor for depressive symptoms after stroke at all measurement times (*B* = 3.32 to 3.97; *p* < 0.01). Physical disability was a risk factor at all measurement times (*B* = −0.09 to −0.03; *p* < 0.05) except 6 months after rehabilitation. Social support was a protective factor (*B* = −2.69 to −1.91; *p* < 0.01) outside the acute phase (*R*^2^ = 0.15–0.39). Intraindividual changes in physical disability and perceived social support were independent predictors of PSD 6 months after the acute phase (*B* = −0.08/−0.14; *p* < 0.01), in addition to status scores on established variables (Δ*R*^2^ = 0.08, *p* < 0.001).

**Discussion:**

History of mental disorder, physical disability, and social support are independent predictors of depressive symptoms in the first year post-stroke, also when considered conjointly. Future studies should control for these variables when investigating new predictors of PSD. In addition, intraindividual changes in known predictors after stroke play a relevant role in the pathogenesis of PSD and should be considered in clinical practice and future research.

## Introduction

Stroke represents the leading cause of acquired disability in adults worldwide (1). In 2017, the absolute number of stroke survivors was estimated to be 9.53 million in the European Union and up to 2047, this number is projected to grow up to 12.11 million, which represents a relative increase of 27% (2). As depression occurs in one third of survivors within the first 5 years after the event (3), the absolute number of people with post-stroke depression (PSD) is expected to rise proportionally. This population faces known adversities like reduced quality of life, cognition, and functional dependence as well as increased mortality compared to stroke survivors without depression (4–7). Therefore, many studies aimed to identify predictors of PSD, which may elucidate the disorder's pathogenesis and facilitate its early identification and subsequent treatment, or even prevention.

Up to now, there is no consensus on a unified assessment method of PSD (8). In the literature, PSD has been defined both as minor and/or major depression according to classification systems like DSM and ICD or by a score above a cut-off on validated depression severity scales (3, 9). The optimal timing for PSD screening also remains unclear (8) as depression may occur up to several years after the stroke and shows a non-linear pattern of emergence (10, 11). Previous evidence underlined the need for early identification of PSD risk factors, which represents a promising strategy to address the timing issue and improve treatment use in PSD, which is characterized by both over- and undertreatment (12, 13). Moreover, meta-analytic evidence demonstrated that psychosocial interventions and pharmacotherapy are efficacious for prevention of depression after stroke, albeit with an increased risk of nausea and bone fracture for pharmacotherapy (14, 15). Hence, preventive interventions should be applied only in groups at high risk of PSD in order to save resources and avoid the occurrence of adverse medication effects in people with low risk. However, these strategies demand reliable evidence on risk factors. Hitherto, several meta-analyses investigated predictors of PSD but their results remain heterogeneous. This challenges researchers and clinicians in concluding which predictors to use for explaining and predicting the development of PSD.

Hackett and Anderson (16) conducted the first systematic review and meta-analysis on predictors of PSD published in 2005, which included 20 prospective longitudinal studies with data of 17,934 stroke survivors and assessing over 100 risk factors across all studies. Representing the most consistently reported risk factors of PSD, physical disability was confirmed as a predictor in 9 out of 11 studies including this variable, stroke severity in five out of five studies, and cognitive impairment in four out of five studies. Moreover, the authors argued to include social support or its counterpart social isolation (in the following, social support is used for both terms) as a relevant predictor in future studies because this variable was confirmed in four out of four studies. However, it must be noted that the interpretation of these results did not consider whether the applied regression analyses were univariable, i.e., investigating one independent variable at a time, or multivariable, which allows to identify the independent effect of each predictor while controlling for the influence of the others variables (17). Considering only the results of multiple regression analyses, physical disability was still confirmed in nine out of 11 studies, but stroke severity and cognitive impairment were only demonstrated in two out of five studies, respectively, and social support in two out of four studies.

About 8 years later, the same research group (18) as well as two further groups (19, 20) updated the results of the first review. Kutlubaev and Hackett (18) revised their previous eligibility criteria to exclude selective samples, which resulted in the exclusion of seven studies from their earlier publication (16). However, they included 10 new samples, resulting in 23 studies with data of 18,374 stroke survivors. The authors confirmed physical disability (significant in 12/13 studies), stroke severity (4/6 studies), and social support (5/5 studies) as predictors. Cognitive impairment was only reported as a significant predictor in two out of three studies. For physical disability, six new studies demonstrated its predictivity in multiple regression analyses. One of the new studies also confirmed social support in multiple regression. None of the new studies investigated stroke severity or cognitive impairment in multiple regression analyses.

Ayerbe et al. (19) addressed this methodological issue by including only observational studies with multiple regression analyses of predictors into their meta-analysis. Moreover, they only considered studies predicting a dichotomous definition of PSD, either by diagnostic classification criteria or a score above a cut-off in a validated scale, while Hackett and Anderson's (16) review and its update (18) also included studies predicting continuous severity of depressive symptoms. Ayerbe et al. (19) assessed ten studies including 16,045 stroke survivors and also confirmed physical disability as the most consistently shown predictor of PSD (4/5 studies) and identified history of mental disorder as a further relevant predictor (3/4 studies). Cognitive impairment and social support were also confirmed in two out of two studies, respectively. Stroke severity, however, was confirmed in none out of three studies. Eventually, De Ryck et al. (20) published a further meta-analysis to update the results by Hackett and Anderson (16). They applied less rigorous inclusion criteria than Ayerbe et al. (19) and identified 24 studies with 9,572 stroke survivors for their analyses. While the authors did not distinguish between univariable and multivariable analyses, they concluded physical disability (14/18 studies), stroke severity (8/10 studies), cognitive impairment (6/15 studies), history of mental disorder (6/10 studies) and lack of social support (5/5 studies) as most consistent predictors of PSD. Although these five predictors were also confirmed in the other meta-analyses, partially even in multiple regression analyses, the summary of these results highlights the lack of a consensus on the predictors of PSD and their selection for studies. Notably, only one single study assessed all of the five established predictors and confirmed associations of physical disability, cognitive impairment, and social support with depressive symptoms 3 months after stroke (21). However, this study had a cross-sectional design and only conducted univariable analyses, which precludes further conclusions on the predictors' independent effects.

While this evidence provides a common basis and suggests that all five risk factors are relevant for PSD, their independent predictive values remain unclear and can only be identified when including all variables in a multiple regression analysis which allows to disentangle the effects of correlated risk factors. For the five established risk factors of PSD, this is of crucial relevance, since they show a substantial overlap among each other (22–24), with the most prominent known overlap between stroke severity and physical disability (25). Thus, this overlap is relevant to account for, in order to assess which of these predictors exert an independent effect. Moreover, controlling for known risk factors provides an important basis to assess which new risk factors of PSD should be introduced (8). While the authors of the meta-analyses argue that the predictive value of new risk factors needs to be compared to the one of known factors (16, 19, 20), there is no empirical validation of these so-called known factors yet.

A further caveat is that previous research primarily assessed risk factors at a single, distinct and to some degree arbitrary point in time after stroke. Such “status scores” are important to draw conclusions about the predictive value of risk factors like e.g., (low) social support or (high) stroke severity. However, this strikingly neglects the marked intraindividual dynamics of impairment and the psychosocial environment after stroke. For example, survivors' disability may ameliorate, reach a plateau or persist without significant changes over the first year after stroke (26, 27). Based on the finding that disability is the most consistent predictor of PSD, these dynamics are likely to influence its development and in turn its relevance and predictive value. The same is true for social support as social relationships may markedly change after stroke (28, 29). Volz et al. (30) demonstrated the relevance of dynamics after stroke as decreasing but not state self-efficacy was a predictor of PSD while controlling for the aforementioned five risk factors. In addition, PSD is proposed to be “mostly associated with the experience and the consequences of stroke itself” [(19), p. 20]. While the debate about psychosocial vs. biological causes of PSD is ongoing (8, 31, 32), the relevant subjective experience of stroke, which includes individual dynamic changes after stroke, is rarely assessed. More specifically, the five established risk factors were concluded based on status scores, which were usually investigated in simple regression models (18–20). Among these, social support represents the only subjective measure self-reported by the stroke survivor, and even this is sometimes assessed *via* the number of people living in a household (33). Therefore, the experienced reality of stroke survivors may be considered more adequately by investigating the (intraindividual) dynamics or in other words the change of known PSD predictors.

To summarize, this paper pursues to address two research gaps: first, we aim to validate the five established risk factors of PSD (history of mental disorder, physical disability, stroke severity, cognitive impairment, and social support) from a multivariable perspective, thus following expert recommendations by assessing the independent predictive value (8). For this, we used two independent prospective longitudinal samples covering the first year after stroke. This allows to determine the independent effects of the predictors previously identified in meta-analyses and thereby conclude, which variables must be considered when investigating new potential risk factors of PSD. Secondly, we aim to examine the additional predictivity of changes in physical disability and social support after stroke as the dynamics in these known risk factors may represent the experience of stroke more adequately than the previously applied state measures.

## Materials and methods

**Figure 1:**
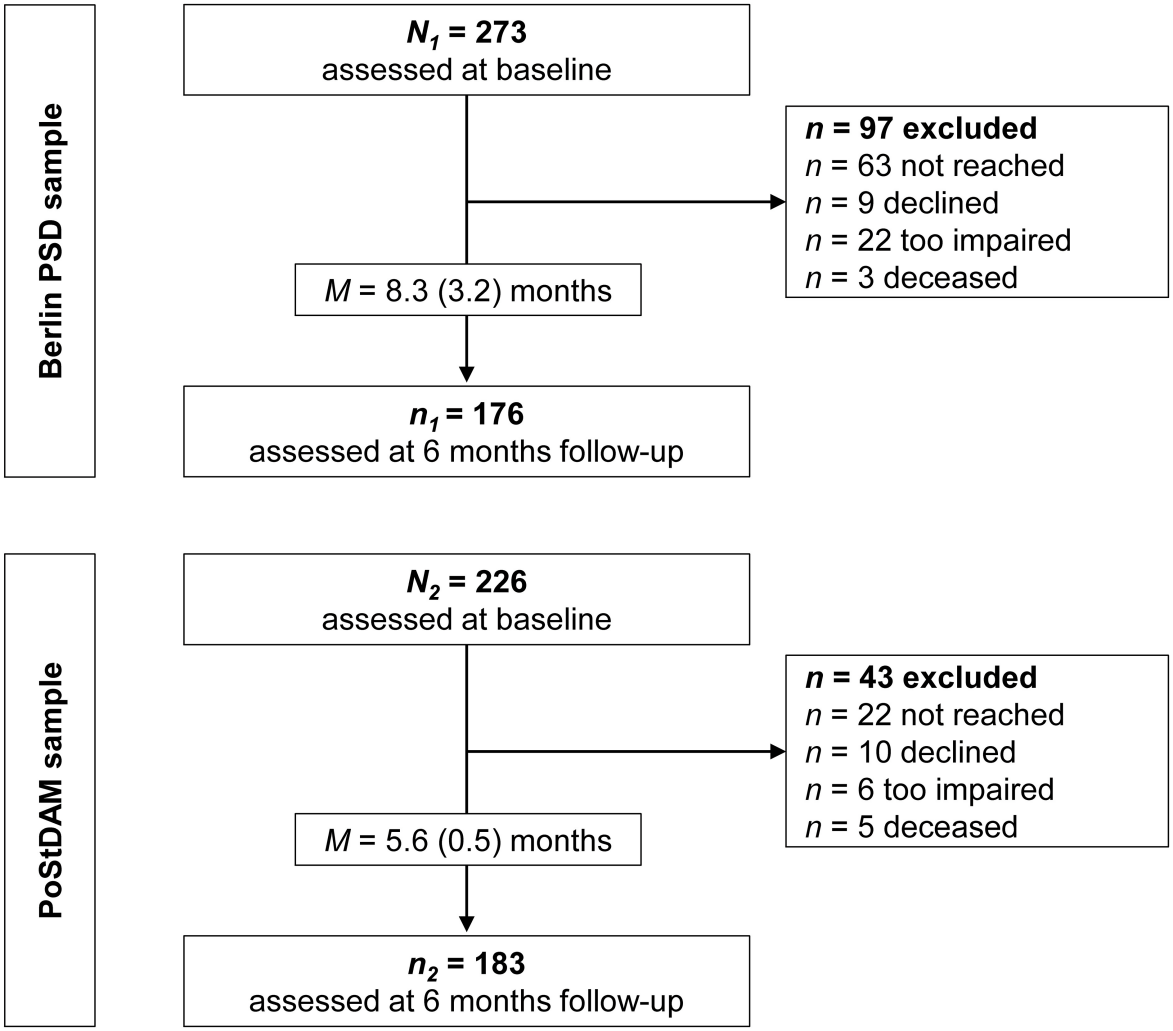
Flow chart for the two study samples. PoStDAM, Post-Stroke Depression: Early Assessment for improved Management; PSD, post-stroke depression.

This analysis is based on the data of two independent prospective longitudinal studies, the Berlin PSD study and the PoStDAM study (=Post-Stroke Depression: Early Assessment for improved Management). Both studies' procedures were previously described in detail (12, 34) and are summarized in the following. Patient flow and reasons for drop-out are reported in Figure 1.

The Berlin PSD study recruited people with ischemic stroke and sufficient language comprehension (Token Test error score < 12) (35) from two inpatient rehabilitation clinics between 2011 and 2016. Participants gave written informed consent to participate in the study. Individuals were excluded if they had a terminal or impairing disease other than stroke (i.e., dementia, neurodegenerative diseases, epilepsy, cancer, AIDS, intellectual disability or other acute life-threatening conditions). Neuropsychologists in the clinics evaluated inclusion and exclusion criteria and referred eligible patients to trained doctoral or master students. Before discharge from the rehabilitation clinics (time since stroke: *M* = 5.9 weeks, *SD* = 2.1), they assessed the following characteristics: history of mental disorder (yes/no; self-report), acute stroke severity (modified National Institutes of Health Stroke Scale [mNIHSS]) (36), physical disability (Barthel Index) (37), cognitive status (Mini Mental Status Examination [MMSE]) (38), and social support (F-SozU K-22) (39). Six months later, participants were followed up *via* telephone interview and the 15-item version of the Geriatric Depression Scale (GDS-15) was used to assess depressive symptoms (40).

The PoStDAM study consecutively recruited people with ischemic stroke (discharge diagnosis), sufficient language comprehension (ascertained by a Aphasia Screening Test comprehension score ≥11 in age ≤ 70 years and a score ≥10 in age ≥71 years) (41), and sufficient cognition (MMSE score ≥ 18) from the stroke unit of an acute hospital between 2018 and 2020. Potential participants were approached *M* = 4.4 days (*SD* = 1.6) after admission and were informed verbally and in written form. In case of visual and/or language impairment, complete written information was read and/or explained to the person. Individuals were included after given informed written consent. In accordance with the Berlin PSD study, participants were excluded if they had another impairing or terminal disease (see above), and trained doctoral or master students conducted the same baseline measures to assess the five established risk factors. Neurologists rated the mNIHSS and nurses completed the Barthel Index. Hereby, social support was assessed by the 14-item version of the F-SozU (F-SozU K-14) (42), opposed to the 22-item version in the Berlin PSD study. However, both versions' scores were determined by dividing the sum score by the number of completed items, which is recommended by the authors and aligns both scales' range (39, 42). Six months later, participants were also followed up *via* telephone interview to assess depressive symptoms using the Patient Health Questionnaire-9 (PHQ-9) (43). Additionally, physical disability (Barthel Index) and social support (F-SozU K-14) were reassessed during the follow-up interviews.

In both studies, antidepressant medication was recorded from discharge reports at baseline and from self-report at follow-ups. The following classes were coded as antidepressant drugs: selective serotonin (norepinephrine) reuptake inhibitors, noradrenergic and specific serotonergic antidepressants, tricyclic and tetracylic antidepressants or monoamine oxidase inhibitors. Diagnosis of depressive disorder (minor or major depression) was ascertained by the Structured Clinical Interview for DSM-IV (44) in the Berlin PSD sample at both measurement times and in the PoStDAM sample at the follow-up. The interview was not conducted at the acute hospital as the diagnosis must not be made briefly after a decisive event like stroke. At both studies' follow-ups, participants were excluded from analysis when the time of the follow-up interview varied more than 12 weeks from the 6 months mark and when they were not reached after six attempts. Both studies were approved by the respective ethics commission at the department of psychology, Humboldt-Universität zu Berlin (Berlin PSD, Reg. No. 2010-13) and at Universität Potsdam (PoStDAM, Application No. 5/2018).

In both samples, multiple linear regression analyses were conducted to predict depressive symptoms at 6 months follow-ups. The included predictors were history of mental disorder (yes/no), stroke severity (mNIHSS), physical disability (Barthel Index), cognitive status (MMSE score </≥ 24), and social support (F-SozU). The MMSE score was dichotomized to align the analysis to recent meta-analytic studies, which proved cognitive impairment to be a risk factor of PSD (18, 20). To examine the additional predictivity of dynamics in physical disability and social support, the analysis in the PoStDAM data were extended by a second regression model, which added the Barthel Index and F-SozU K-14 difference scores between baseline and follow-up to the five other predictors. Difference in model fit (residual sum of squares = *RSS*) between the two models was tested for significance using an ANOVA. To examine the relationship of changes in depressive symptoms with changes in physical disability and social support, Pearson correlations of the PHQ-9 change scores (difference between follow-up and baseline) and the physical disability and the social support change scores were calculated, respectively. To further investigate the role of direction in change, i.e., if increase or decrease of physical disability and social support represents the relevant change, a further multiple linear regression analysis was conducted to predict depressive symptoms after 6 months in the PoStDAM data. The five established variables served as predictors as well as four dummy-coded direction of change variables (increase and decrease in physical disability and social support, respectively). To compute these variables, all values < −1 standard deviation were defined as decrease and all values > 1 standard deviation as increase in the difference scores.

Selectivity of drop-out and differences between the two study samples were examined by applying Welch's *t*-tests for independent samples for continuous (45) and χ^2^-tests for dichotomous variables. Significance level was set at α = 0.05 and Bonferroni-adjusted to correct for multiple comparisons. All calculations were performed using the R environment Version 4.2.1 (46).

## Results

Detailed descriptive statistics for both samples, all measurement points and comparisons between both samples are illustrated in Table 1. Correlations among all predictors and their variance inflation factors are shown in Supplementary Table 1 (Berlin PSD) and Supplementary Table 2 (PoStDAM).

**Table 1:** Both samples' descriptive statistics and their comparison at baseline and follow-up.

	**Berlin PSD**						**PoStDAM**						**Berlin PSD vs. PoStDAM** ***^**3**^***			
	**Baseline (*****N*** = **273)**			**Follow-up (*****n*** = **176)**			**Baseline (*****N*** = **226)**			**Follow-up (*****n*** = **183)**			**Baseline**		**Follow-up**	
	***n*** **(%)**			***n*** **(%)**			***n*** **(%)**			***n*** **(%)**			χ^2^	***p***	χ^2^	***p***
Gender (female)	112 (41.0)			82 (41.8)			107 (47.3)			83 (45.4)			1.76	0.185	0.09	0.758
History of mental disorder (yes)	36 (13.2)			24 (13.6)			61 (27.0)			51 (27.9)			13.78	**< 0.001**	14.38	**< 0.001**
First-ever stroke (yes)	182 (72.8)			118 (67.0)			182 (80.5)			149 (81.4)			1.90	0.169	4.23	0.040
Lesion location													0.47	0.789	0.39	0.824
Right hemisphere	116 (47.2)^a^			77 (47.2)^b^			88 (41.9)^c^			64 (37.9)^d^						
Left hemisphere	100 (40.7)^a^			66 (40.5)^b^			93 (44.3)^c^			81 (47.9)^d^						
Other^*^	30 (12.2)^a^			20 (12.3)^b^			29 (13.8)^c^			24 (14.2)^d^						
Cognition impaired (MMSE < 24)	19 (7.0)			10 (5.7)			35 (15.5)			25 (13.7)			8.20	0.004	5.45	0.020
Antidepressant medication (yes)	59 (21.7)^e^			26 (16.5)^f^			24 (10.6)			14 (7.7)			3.58	0.058	5.44	0.020
Depressive disorder (yes)	94 (34.4)			53 (30.1)			–			32 (17.5)			–	–	7.23	0.007
	**M**	**SD**	**Range**	**M**	**SD**	**Range**	**M**	**SD**	**Range**	**M**	**SD**	**Range**	***t***	***p***	***t***	***p***
Age (years)	63.9	10.9	42–92	64.2	10.5	42–90	70.8	12.8	24–97	70.0	12.5	24–93	−6.29	**< 0.001**	−4.72	**< 0.001**
Education (years)	11.3	3.1	8–18	11.5	3.1	8–18	13.8	3.6	6–23	13.9	3.6	6–22	−8.20	**< 0.001**	−6.89	**< 0.001**
MMSE	27.8	2.6	15–30	28.0	2.5	17–30	26.3	2.8	18–30	26.6	2.7	18–30	6.11	**< 0.001**	5.18	**< 0.001**
mNIHSS	3.6	3.2	0–18	3.6	3.3	0–18	2.5	3.0	0–27	2.2	2.5	0–13	4.04	**< 0.001**	4.42	**< 0.001**
Barthel Index	86.8	18.3	25–100	87.6	18.5	25–100	74.1	28.4	0–100	80.2	23.9	5–100	5.72	**< 0.001**	3.29	**0.001**
ΔBarthel Index	–	–	–	–	–	–	–	–	–	13.7	21.6	−30 to 85	–	–	–	–
F-SozU^1^	4.3	0.7	1.2–5	–	–	–	4.4	0.6	2.07–5	4.4	0.6	2–5	−2.39	0.017	−2.11	0.035
ΔF-SozU	–	–	–	–	–	–	–	–	–	−0.9	0.5	−2 to 2.1	–	–	–	–
Depressive symptoms (GDS-15/PHQ-9)^2^	3.7	3.6	0–15	3.7	3.7	0–15	5.6	4.6	0–27	5.6	4.8	0–27	−5.00	**< 0.001**	−5.49	**< 0.001**

1 Berlin PSD: FsozU-K22; PoStDAM: FsozU-K14; for comparability between the 14 and 22-item version, total mean scores divided by the number of items were compared [cf. Fydrich et al. (40)].

2 Berlin PSD: GDS-15; PoStDAM: PHQ-9.

3 Bonferroni-adjusted significance level of p = 0.0038 was used. Significant p-values are marked as bold.

a Data of n = 246,

b n = 163,

c n = 210,

d n = 169,

e n = 272,

f n = 158 participants available.

* Bilateral and/or subcortical location.

F-SozU, Social Support Questionnaire; GDS-15, Geriatric Depression Scale-15; MMSE, Mini Mental State Examination; mNIHSS, modified National Institutes of Health Stroke Scale; PHQ-9, Patient Health Questionnaire-9; PoStDAM, Post-Stroke Depression: Early Assessment for improved Management; PSD, post-stroke depression.

In the Berlin PSD study, *N*_1_ = 273 participants were recruited at baseline and *n*_1_ = 176 were followed-up after 6 months (drop-out rate = 35%). Drop-out analysis showed no significant differences in demographics (age, gender, education) and clinical variables including the five established risk factors and depressive symptoms between people participating at follow-up and drop-outs. In the PoStDAM study, *N*_2_ = 226 were recruited at baseline and *n*_2_ = 183 were followed-up 6 months later (drop-out rate = 19%). Drop-out analyses revealed that people participating at the follow-up showed lower physical disability at baseline than people not participating (*p* < 0.001). No differences were found in demographic and clinical variables including the other established risk factors and depressive symptoms. Figure 1 displays the attrition in both samples including reasons for drop-out.

**Figure 2:**
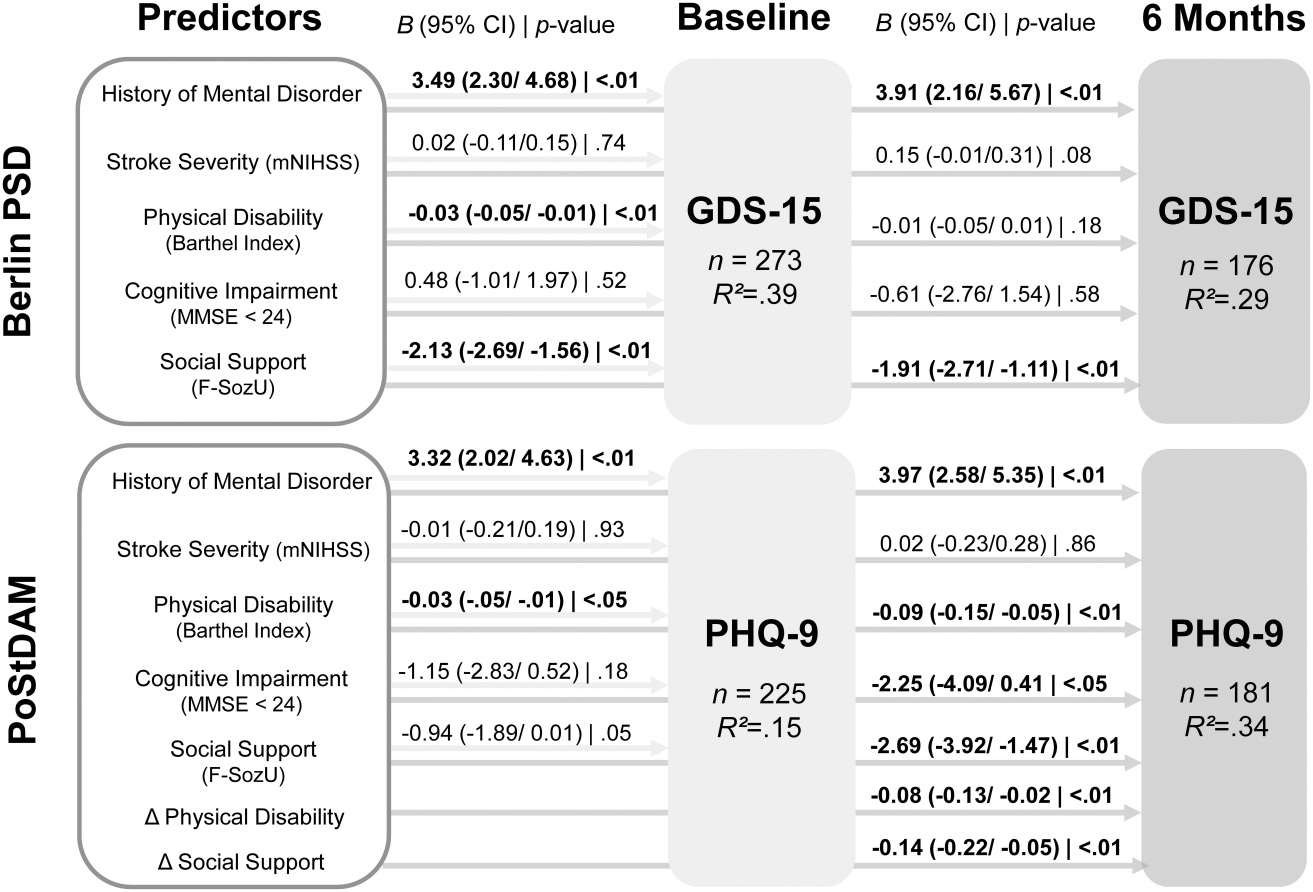
Results of multiple linear regression analyses predicting depressive symptoms at baseline and 6 months follow-up in both samples. Beta coefficients for PoStDAM 6 months refer to the regression model with all seven predictors. CI, confidence interval; F-SozU, Social Support Questionnaire; GDS-15, Geriatric Depression Scale, 15 item version; MMSE, Mini Mental State Examination; mNIHSS, modified National Institutes of Health Stoke Scale; PHQ-9, Patient Health Questionnaire-9; PoStDAM, Post-Stroke Depression: Early Assessment for improved Management; PSD, post-stroke depression. *B* = unstandardized beta coefficient, Δ = difference between follow-up and baseline; *R*^2^ = adjusted *R*^2^.

Figure 2 displays the results of the regression analyses predicting depressive symptoms in both samples at baseline and the 6-month follow-up. The exact *t*- and *p*-values for each predictor are shown in Supplementary Table 3. For the PoStDAM sample, the regression model that includes only the five established risk factors to predict depressive symptoms at 6 months (not shown in Figure 2) revealed history of mental disorder (*B* = 4.26, 95% CI = 2.83/5.68; *t* = 5.89; *p* < 0.001), physical disability (*B* = −0.03, 95% CI = −0.06/−0.01; *t* = −2.42; *p* = 0.016), cognitive impairment (*B* = −2.47, 95% CI = −4.39/−0.55; *t* = −2.54; *p* = 0.012), and social support (*B* = −1.93, 95% CI = −3.10/−0.75; *t* = −3.23; *p* = 0.001) as predictors (*R*^2^ = 0.26). Stroke severity showed no association (*B* = 0.12, 95% CI = −0.14/0.39; *t* = 0.92; *p* = 0.357).

Taken together, history of mental disorder was confirmed as a predictor in both independent samples at both measurement points and physical disability was confirmed at all measurement points but 6 months after inpatient rehabilitation (Berlin PSD, follow-up). Social support was confirmed as a predictor at all measurement points but the acute phase (PoStDAM, baseline), however social support almost reached significance here (*p* = 0.053). Stroke severity did not predict depressive symptoms at any measurement point in multiple regression analyses. Cognitive impairment only predicted depressive symptoms 6 months after the acute phase (PoStDAM, follow-up). We re-ran our analyses with a continuous MMSE score to examine whether the dichotomization limited statistical power. This procedure demonstrated that cognitive impairment was associated with baseline depression (*B* = −0.17, 95% CI = −0.32/−0.03; *t* = −2.31; *p* = 0.022) in the rehabilitation-based sample while the overall pattern of the other risk factors did not change. However, the continuous cognition score showed no significant association with baseline depression in the hospital-based or follow-up depression in both samples.

Notably, considering difference scores to capture the dynamics of predictors showed that change in assessed physical disability and perceived social support significantly predicted depressive symptoms 6 months after the acute phase (PoStDAM, follow-up) while controlling for the established risk factors (cf. Figure 2). Most importantly, the addition of the two difference scores significantly increased the model's fit, emphasizing their additional predictive value [*RSS*_1_ = 3,114.8, *RSS*_2_ = 2,818.9, Δ*R*^2^ = 0.08, *F*_(2,173)_ = 9.08, *p* < 0.001].

In bivariate correlation analyses, the change in physical disability showed no significant association with change in depressive symptoms (*r* = −0.08, *p* = 0.296), while change in social support was significantly correlated with change in depressive symptoms (*r* = −0.28, *p* < 0.001). Further analysis assessing the direction of change *via* four dummy-coded variables yielded that physical disability decreased in *n* = 12 (6.6%) and increased in *n* = 27 (14.8%), while social support decreased in *n* = 27 (14.8%) and increased in *n* = 22 (12.0%) participants. In the multiple regression analysis adding these dummy variables (*R*^2^ = 0.31), history of mental disorder (*B* = 3.92, 95% CI = 2.52/5.32; *t* = 5.49; *p* < 0.001), physical disability (*B* = −0.05, 95% CI = −0.09/−0.01; *t* = −2.61; *p* = 0.009), cognitive impairment (*B* = −2.50, 95% CI = −4.36/−0.63; *t* = −2.62; *p* = 0.009), and social support (*B* = −2.63, 95% CI = −4.02/−1.25; *t* = −3.72; *p* < 0.001) remained significant predictors. For physical disability, neither a decrease (*B* = 1.41, 95% CI = −1.09/3.90; *t* = 1.11; *p* =0.269) nor an increase (*B* = −1.09, 95% CI = −3.43/1.26; *t* = −0.91; *p* = 0.364) significantly predicted depressive symptoms. However, a decrease in social support significantly predicted depressive symptoms (*B* = 1.95, 95% CI = 0.19/3.70; *t* = 2.17; *p* = 0.031), while an increase in social support showed no significant predictive effect (*B* = −2.02, 95% CI = −4.32/0.28; *t* = −1.72; *p* = 0.087).

## Discussion

The present study aimed to address two research gaps regarding the predictive value of well-established risk factors of PSD: first, drawing from recent meta-analytic evidence, our study assessed the five established risk factors (history of mental disorder, stroke severity, physical disability, cognitive impairment, and social support) from a multivariable perspective during the first 6 months after stroke in two independent stroke samples from two different settings (hospital vs. rehabilitation). With this, we do not only apply relevant (methodological) recommendations (8), but assess the independent predictive value of each risk factor while accounting for the influence of the others. Due to the substantial overlap between the established risk factors (cf. Supplementary Tables 1, 2), this procedure further disentangles the influence of the predictors and sharpens evidence about their relevance for the pathogenesis of PSD. Second, we introduced a dynamic perspective of certain risk factors, which accounts for the *intraindividual changes* of these risk factors during the first 6 months after stroke. With this, we extended evidence about the predictive value by acknowledging the known dynamics of clinical and psychosocial features within the first year after stroke (10, 26, 27). Our multiple regression analyses concordantly showed that the presence of previous mental disorder, higher physical disability and lower social support are independent risk factors for PSD. Notably, these three factors were relevant for various time points after stroke, ranging from baseline assessments within the acute setting, to the completion of inpatient rehabilitation up to 6 months after acute care and rehabilitation. In our two samples, stroke severity and cognitive impairment—albeit representing established risk factors—were univariably associated with PSD (cf. Supplementary Tables 1, 2), but not consistently predictive for PSD when included in multiple regression analysis. For stroke severity, this supports previous meta-analytic evidence (16, 18) suggesting that its influence fades when physical disability is taken into account, due to the high correlation between these two factors (PSD Berlin: *r* = −0.39; *p* < 0.01; PoStDAM: *r* = −0.38; *p* < 0.01; cf. Supplementary Tables 1, 2). It must be noted that the integration of two different recruitment settings and the varying time intervals covered within stroke rehabilitation support the generalizability of these results.

From a more fine-grained perspective, we found that physical disability was not predictive for the follow-up assessment in the rehabilitation-based Berlin PSD sample, in contrast to the hospital-based PoStDAM sample. Comparison between our two samples showed that participants in the PoStDAM sample showed significantly higher physical disability, which is an expected bias considering that the PoStDAM study recruited stroke survivors a few days after the event, a markedly earlier point than before discharge from rehabilitation in the Berlin PSD study. Moreover, the Barthel Index score in the Berlin PSD sample was skewed with a median of 95 points (compared to the mean of 85.72) and showed significantly lower variance, *F*_(268/225)_ = 0.46, *p* < 0.001. This means that physical disability was low and less varying in the Berlin PSD sample which probably limited the relevance of this risk factor for the rehabilitation-based sample. In addition, the adequacy of the Barthel Index may be questioned when the aim is to assess all aspects of functional impairment that are relevant to everyday life 6 months after inpatient stroke rehabilitation. While the Barthel Index assesses impairment in basic tasks, capturing more complex tasks might represent a more relevant risk factor (e.g., Instrumental Activities of Daily Living) (47), not only for stroke survivors experiencing less physical disability but also for younger stroke survivors which do not only report less physical impairment but also struggle with vocational reintegration after stroke (48).

A similar pattern emerged for perceived social support, where our results showed no significant association at baseline in the hospital-based PoStDAM sample, in contrast to the Berlin PSD sample. From a methodological standpoint, this could be due to a power issue given the *p*-value of 0.053. Nevertheless, participants were assessed about 4 days after stroke, which might not allow stroke survivors to individually perceive markedly different levels of social support, since they have limited contact to their relatives in the acute setting. This might imply that the answers given reflect the perception of pre-stroke social support in contrast to the Berlin PSD sample where patients were assessed at later stages—about 6 weeks after stroke. Interestingly, perceived social support *decreased* in the hospital-based sample and both baseline and change in social support was predictive for PSD after 6 months. Given that baseline social support was not different between the two samples, but the range was higher for the rehabilitation-based sample, this might further support the idea that differences in social support and their effect on PSD need time to manifest in the perception of stroke survivors.

Cognitive impairment was not predictive for PSD at baseline in both samples when we controlled for the other established risk factors. Since cognitive impairment was dichotomized (score </≥ 24) to align the analysis to recent meta-analytic studies (18, 20) we re-ran our analyses with the continuous score, which demonstrated that cognitive impairment was only associated with baseline depression in the rehabilitation-based sample. In comparison, the dichotomous cognition score showed an association only with follow-up depression in the hospital-based sample. Moreover, baseline cognitive impairment was lower in the rehabilitation-based compared to the hospital-based sample (Table 1). Therefore, the continuous score may be more adequate for samples with lower cognitive impairment and suggest that the cut-off for cognitive impairment applied in previous studies is not ideal and decreases statistical power. In addition, as for the Barthel Index, instruments assessing more complex cognition than a simple global screening like the MMSE may be more adequate to predict mood alterations after stroke (49).

Regarding our second aim—assessing the relevance of change scores as predictors for later PSD, our results showed that change in physical disability and social support were independent risk factors, even when all five established risk factors were controlled for. Most importantly, adding the two difference scores to the initial model significantly increased its fit, emphasizing their additional predictive value. Of note is that our multiple regression analysis addresses the fact that baseline and change scores share common variance. In other words, perceived change in social support and physical disability can predict later PSD, even when we controlled for the respective baseline scores. Our results showed that decreasing social support and increasing physical disability was associated with higher depressive symptoms. Moreover, decreasing social support was associated with *increasing* depressive symptoms within 6 months after acute stroke, while no association was found between time-dependent changes in physical disability and changes in depressive symptoms. These results suggest that post-stroke disturbances in the psychosocial environment of survivors are strongly connected to the emergence of depressive symptoms. This is in line with the hypothesis of ecosystem focused therapy that the so-called psychosocial storm following stroke plays a crucial role in the pathogenesis of PSD (50). However, the association between changes in physical disability and depressive symptoms is only detected when other risk factors are controlled for. This may trace back to high shared variance between disability and the other risk factors, opposed to social support (cf. Supplementary Table 2), and highlights the relevance of including the other risk factors in multivariable models. Nevertheless, these results need to be interpreted cautiously due to limited power, especially in the analyses using the dummy-coded variables.

While our study relies on two independent stroke samples from different settings and robust and cautious methodological approaches, we wish to acknowledge the following limitations. First, our two samples used two different instruments for assessing PSD, i.e., GDS-15 for the Berlin PSD sample, and the PHQ-9 for the PoStDAM sample. Although both instruments are amongst the most established and valid tools to assess PSD (18, 51), the GDS might capture other aspects of PSD due to omitting somatic items in contrast to the PHQ-9. Including somatic items in the assessment of PSD has been controversially debated and draws its controversy from etiological and phenomenological standpoints (8, 10, 32). From a more phenomenological standpoint, a recent high-quality study has demonstrated that symptom profiles of depression between people with and without stroke do not differ in the occurrence of specific symptoms, suggesting that the inclusion of somatic items does not bias assessed frequency and severity (52). Nevertheless, the different instruments used in our study might limit the comparability of our results. Second, the inclusion criteria differed between studies with the PoStDAM study having an additional inclusion criterion of sufficient cognition (MMSE ≥ 18). This may partially explain the heterogenous results regarding cognitive impairment between the two studies. However, the PoStDAM sample showed higher cognitive impairment despite the additional inclusion criterion on cognition. Third, people who participated in the follow-up of the PoStDAM sample showed lower physical disability at baseline, which is a common finding in longitudinal stroke studies but might have biased results (8). Moreover, the two samples differed in characteristics with people in the acute setting (PoStDAM) being older, having higher education, higher physical disability, lower stroke severity, and a higher proportion of pre-stroke mental disorders compared to the rehabilitation-based sample (Berlin PSD). Since some of these differences are to be expected due to the different settings (age, disability, and stroke severity), this might explain some heterogeneity in the reported results. On a side note, while higher physical disability and simultaneously lower stroke severity in the acute phase may appear contradictory, higher stroke severity (e.g., deficits in perception or language) probably hindered stroke survivors from participating in the PoStDAM study while physical disability usually did not interfere with study participation. Moreover, stroke severity was an acute phase measure in both studies and therefore, interfered less with participation in the rehabilitation-based sample. The proportion of depressive disorders in the rehabilitation-based sample (30–34%) was comparable to the frequencies reported for rehabilitation-based samples in a recent meta-analysis (0–1 months: 33%, 95% CI = 23/34%; 6–9 months: 44%, 95% CI = 29%/59%) (3). In the acute sample 6 months after stroke however, depression prevalence (17.5%) was lower than the frequency reported in the meta-analysis for hospital-based studies 6–9 months after stroke (26%, 95% CI = 22%/30%) (3). This may be due to heterogeneity in assessment methods and times as none of the studies applied a structured interview 6 months after hospital. The most comparable study applied a structured interview 4 months after the hospital and reported a similar prevalence of 19% (95% CI = 12%/27%) (53). Fourth, to align our research with meta-analytic evidence, cognitive impairment was dichotomized, which might have limited statistical power. To tackle this problem, we performed *post-hoc* analyses assessing cognitive impairment from a continuous perspective. Lastly, while we introduce and argue for a dynamic perspective due to growing evidence on a time-dependent component of relevant risk factors (10, 32), future studies should further assess heterogeneity in intraindividual changes over time. This also extends to investigating predictors of time-varying depressive symptoms as previous studies have demonstrated distinguishable trajectories of depression after stroke (54, 55).

From a clinical point of view, our results suggest that for the risk assessment during the acute and post-acute setting, clinicians might benefit from not only considering the static level/severity of risk factors, but also its development over time in order to improve the early identification of people at risk for PSD. While PSD is still considered as underdiagnosed and undertreated (8, 56–58), this would call for repeated assessment of potential PSD risk factors, which might be even more difficult to implement in clinical routines than one-time assessments. Nevertheless, given the relevance for PSD prevention and treatment, both from a clinical and economic perspective, our results emphasize the potential benefit of change scores in the prediction of PSD. Moreover, our results demonstrate that changes in social support may be a target for psychosocial prevention and/or treatment of PSD, which is in line with the approach of ecosystem focused therapy for PSD (50).

To conclude, our study is the first to simultaneously assess all five established risk factors in two independent stroke samples from different settings. Our results concordantly confirmed history of mental disorder, physical disability, and social support as independent risk factors during the first year of stroke. Our results extend previous recommendations (8) and suggest that these factors should be taken into account when assessing other PSD risk factors. Stroke severity appears to have no additional predictive value, when controlling for the other known risk factors, which supports and extends previous findings (18–20). Moreover, we found that stroke survivors' mental health is affected by the dynamics of their physical rehabilitation and psychosocial changes, adding further need to take a time-dependent perspective when investigating the pathogenesis of PSD (10, 59, 60). Such a time-sensitive perspective might elucidate the heterogenous results of previous research and contribute to a better understanding of PSD and its development.

## Data availability statement

The raw data supporting the conclusions of this article will be made available by the authors, without undue reservation.

## Ethics statement

The studies involving human participants were reviewed and approved by Humboldt-Universität zu Berlin, Department of Psychology and Universität Potsdam. The patients/participants provided their written informed consent to participate in this study.

## Author contributions

SL and MV each planned one of the studies and were responsible for their implementation. SL developed the research questions for the conjoint analysis. KW supervised the planning and implementation of both studies. MS advised the planning and implementation of the PoStDAM study. All authors contributed to and approved the final manuscript.
