# Supplementary material for: Predictors of post-stroke depression: Validation of established risk factors and introduction of a dynamic perspective in two longitudinal studies
Source: Front Psychiatry. 2023 Feb 13;14:1093918. doi: 10.3389/fpsyt.2023.1093918 (PMC9969555; doi:10.3389/fpsyt.2023.1093918)
Supplement: Supplementary file 1 [file Table_1.pdf]

Online Supplement ESM 1.

Means, standard deviations, and correlations with confidence intervals for Berlin PSD sample at baseline.

	M	*SD*	VIF	1	2	3	4	5
1. History of mental disorder	0.13	0.34	1.14					
2. Stroke severity	3.64	3.25	1.25	-.03				
3. Physical disability	86.82	19.27	1.31	.11<br>[-.01, .23]	-.39**<br>[-.49, -.28]			
4. Cognitive impairment (MMSE < 24)	0.07	0.26	1.14	-.10<br>[-.22, .02]	.22**<br>[.09, .33]	-.19**<br>[-.31, -.08]		
5. Social support	4.30	0.69	1.22	-.27**<br>[-.38, -.16]	.01<br>[-.11, .14]	.20**<br>[.08, .31]	-.18**<br>[-.29, -.06]	
6. Cognition (MMSE score)	27.81	2.65	-	.08<br>[-.04, .20]	-.23**<br>[-.34, -.11]	.29**<br>[.17, .39]	-.77**<br>[-.81, -.71]	.23**<br>[.11, .34]

Note. MMSE = Mini Mental Status Examination, VIF = variance inflation factor. VIFs were calculated based on the regression model using the dichotomous MMST score. Values in square brackets indicate the 95% confidence interval for each correlation.
* indicates $p < .05$. ** indicates $p < .01$.

Online Supplement ESM 2.

Means, standard deviations, and correlations with confidence intervals for the PoStDAM sample at baseline.

	M	*SD*	VIF	1	2	3	4	5	6	7
1. History of mental disorder	0.27	0.44	1.11							
2. Stroke severity	2.47	3.05	1.16	-.09						
				[-.22, .04]						
3. Physical disability	74.11	28.41	4.05	.12	-.38**					
				[-.01, .25]	[-.49, -.27]					
4. Cognitive impairment (MMSE < 24)	0.15	0.36	1.13	-.04	.19**	-.35**				
				[-.17, .09]	[.06, .32]	[-.46, -.23]				
5. Social support	4.44	0.61	1.31	-.21**	.13*	.01	-.04			
				[-.34, -.09]	[.00, .26]	[-.12, .14]	[-.17, .09]			
6. ΔPhysical disability	13.68	21.55	3.74	-.11	.15*	-.84**	.25**	-.02		
				[-.25, .04]	[.01, .29]	[-.88, -.80]	[.11, .38]	[-.17, .12]		
7. ΔSocial support	-1.38	7.64	1.22	-.05	-.07	-.01	.04	-.40**	.01	
				[-.20, .09]	[-.22, .07]	[-.16, .13]	[-.10, .19]	[-.51, -.27]	[-.14, .15]	
8. Cognition (MMSE score)	26.30	2.82	-	.09	-.23**	.41**	-.77**	.05	-.23**	-.01
				[-.04, .22]	[-.35, -.11]	[.30, .52]	[-.82, -.71]	[-.08, .18]	[-.36, -.09]	[-.15, .14]

Note. MMSE = Mini Mental Status Examination, VIF = variance inflation factor. VIFs were calculated based on the regression model using the dichotomous MMST score. Values in square brackets indicate the 95% confidence interval for each correlation.
* indicates $p < .05$. ** indicates $p < .01$.

Online Supplement ESM 3.

Exact *t*- and *p*-values for predictors in multivariable regression analyses shown in Figure 2.

	Berlin PSD				PoStDAM			
	Baseline		Follow-Up		Baseline		Follow-Up	
	t	*p*	*t*	*p*	*t*	*p*	*t*	*p*
History of mental disorder	10.27	<.001	4.37	<.001	4.99	<.001	5.62	<.001
Stroke severity	0.32	.747	1.79	.075	-0.08	.933	0.18	.855
Physical disability	-2.84	.005	-1.12	.267	-2.44	.016	-3.86	<.001
Cognitive impairment (MMSE < 24)	0.63	.532	-0.56	.577	-1.35	.178	-2.39	.018
Social support	-7.33	<.001	-4.67	<.001	-1.94	.053	-4.32	<.001
ΔPhysical disability	-	-	-	-	-	-	-2.83	.005
ΔSocial support	-	-	-	-	-	-	-3.21	.002

Note. MMST = Mini Mental Status Test, Δ = difference score: follow-up – baseline.
